# Phthalocyanine-based mouth gel as an adjunct to non-surgical periodontal treatment: a randomized controlled trial

**DOI:** 10.1007/s00210-026-05517-0

**Published:** 2026-06-06

**Authors:** Sebastião Orestes Pereira Neto, Pedro Luiz Rosalen, João Vitor da Cruz Pegoraro, Isadora Stella Souza e Andrade, Ana Luiza Marques Reis, Isabela Silva Costa, Gabriela Tiburcio Silva, Guilherme José Pimentel Lopes de Oliveira, Fabiano Vieira Vilhena, Masaharu Ikegaki, Suzane Cristina Pigossi, Leandro Araújo Fernandes, Marcelo Franchin

**Affiliations:** 1 School of Dentistry, Federal University of Alfenas (Unifal-MG), Alfenas, Minas Gerais Brazil; 2 School of Pharmaceutical Sciences, Federal University of Alfenas (Unifal-MG), Alfenas, Brazil; 3 Department of Periodontology and Implantology, School of Dentistry, Federal University of Uberlandia - UFU, Uberlandia, Minas Gerais Brazil; 4 TRIALS – Oral Health & Technologies, Bauru, Brazil; 5 Graduate Program in Biological Sciences, Federal University of Alfenas (Unifal-MG), Alfenas, Brazil

**Keywords:** Periodontitis, Anti-inflammatory agents, Chlorhexidine

## Abstract

This study aimed to evaluate the effect of topical application of a mouth gel formulation containing iron tetracarboxyphthalocyanine (FeTcPc) during non-surgical periodontal treatment. Forty-seven individuals with periodontitis underwent a standard periodontal treatment (scaling and root planing), followed by adjuvant therapy according to the = groups: Vehicle Group (*n* = 15), receiving local application of the FeTcPc gel vehicle; Chlorhexidine (CLX) Group (*n* = 16), receiving local application of 0.12% chlorhexidine gel; and FeTcPc Group (*n* = 16), receiving local application of 1% FeTcPc-containing gel. Clinical evaluations were performed at baseline and after 45 days. No statistically significant differences were observed between the study groups in any periodontal clinical parameters at baseline or 45 days after treatment. In the intragroup analysis, the mean probing depth (PD) and the percentage of sites with PD ≤ 3 mm and PD = 4–5 mm showed a statistically significant reduction at 45 days in both the vehicle and FeTcPc groups (*p* < 0.05). The percentage of sites with bleeding on probing and the plaque index demonstrated a statistically significant reduction after 45 days in all groups (*p* < 0.05). The use of FeTcPc mouth gel as an adjunct to non-surgical periodontal therapy did not improve the clinical periodontal parameters compared to CLX or the vehicle group in this study.

## Introduction

Periodontitis is a chronic inflammatory condition characterized by the progressive destruction of periodontal tissues, significantly impacting both oral and systemic health (Papapanou et al. 2018). This condition is associated with complications such as tooth loss and malnutrition, and it also serves as an aggravating factor for comorbid disorders, including type 2 diabetes, driven by the low-grade systemic inflammation associated with periodontitis (Hajishengallis 2022). Beyond its health implications, periodontitis also imposes a substantial global economic burden, with costs related to treatment and productivity losses estimated at nearly $79 billion annually (Collaborators 2018).

Addressing the multifactorial nature of periodontitis requires a combination of strategies to halt disease progression. These include reducing bacterial biofilms through surgical or non-surgical therapy, providing oral hygiene instruction with patient motivation, and managing modulating factors such as systemic diseases (Kwon et al. 2021). Scaling and root planing (SRP) remains the gold standard in non-surgical periodontal therapy. However, mechanical therapy alone may fail to eliminate pathogenic bacteria located in soft tissues and areas inaccessible to periodontal instruments, such as furcation areas, root concavities, interproximal areas, and sites with deeper pockets (Matia et al. 1986; Adriaens et al. 1988).

To address these challenges and improve treatment outcomes, adjuvant therapies have emerged as promising complementary approaches, reducing the need for invasive procedures (Gegout et al. 2023). These therapies involve the use of physical or chemical agents, host modulators, and antimicrobials, which can be administered locally or systemically (Smiley et al. 2015). Adjuvant agents offer several benefits, including enhanced control of bacterial biofilms, modulation of the inflammatory response, and promotion of healing and regeneration of periodontal tissues (Gomes et al. 2020).

Iron tetracarboxyphthalocyanine (FeTcPc) is a synthetic phthalocyanine derivative that has demonstrated promising activity in modulating inflammatory processes and is low-toxicity (Breseghello et al. 2025). A previous study demonstrated that the pure phthalocyanine compound effectively reduced tumor necrosis factor-alpha (TNF-α) production in activated macrophage cultures by inhibiting the activation of the transcription factor nuclear factor kappa B (NF-κB) (Breseghello et al. 2025). In the same study, the researchers developed a mouth gel containing FeTcPc and found that its topical application significantly reduced alveolar bone loss in mice with ligature-induced periodontitis. This effect was attributed to downregulation of inflammatory genes (e.g., Tnfα and p65 signaling) in the gingival tissue. In this context, the FeTcPc-containing mouth gel is a promising alternative for adjunctive periodontal therapy.

Thus, this study proposes investigating the effects of a mouth gel containing FeTcPc as a novel adjuvant therapeutic approach in the treatment of non-surgical periodontitis. The development of this mouth gel enables topical application of the therapeutic agent, aiming to maximize its efficacy while minimizing undesirable systemic effects. Comprehensive studies evaluating this new treatment not only expand the therapeutic arsenal but also offer new perspectives for managing periodontitis, a significant global health challenge.

## Methods

### Trial design

This double-blind, randomized, placebo-controlled trial was conducted from January 2023 to December 2024. The study was approved by the Research Ethics Committee of the Alfenas Federal University (CAAE: 39624820.6.0000.5142) and was carried out in compliance with the Helsinki Declaration of 1975, as revised in 2013 (World Medical 2013). The complete study protocol was registered at ClinicalTrials.gov (NCT06731777), and the study was reported in accordance with the CONSORT 2010 statement (Schulz et al. 2010).

The selected individuals (*n* = 47) were randomly divided into three groups: Vehicle group (*n* = 15): toothbrushing with toothpaste and the local application of the FeTcPc mouth gel vehicle (without the active ingredients); Chlorhexidine (CLX) group (*n* = 16): toothbrushing with toothpaste and local application of 0.12% CLX mouth gel and FeTcPc group (*n* = 16): toothbrushing with toothpaste and local application of 1% FeTcPc (C₃₂H₁₆N₈O₈Fe, molecular weight: 744.42 g/mol, purity > 90%) mouth gel (PHTALOX®, TRIALS – Oral Health & Technologies, Bauru, Brazil). Clinical evaluations (plaque index, probing depth, bleeding on probing, clinical attachment level, and mobility test) were made at baseline (day 0) and post-treatment (day 45).

### Participants

Forty-seven individuals with periodontitis were recruited from the Periodontics Clinic of the School of Dentistry at Alfenas Federal University. Each patient was informed about the study’s nature and signed a consent form. The study inclusion criteria were as follows: adult individuals (≥ 18 years old) of both sexes diagnosed with periodontitis stages II to IV (moderate to severe) and grades A and B, presenting at least two non-adjacent interproximal sites with a probing depth ≥ 5 mm, clinical attachment level ≥ 3 mm, and bleeding on probing. Participants were required to have at least 15 teeth, excluding third molars.

Exclusion criteria included medical conditions requiring antibiotic prophylaxis or those potentially impacting treatment outcomes, history of periodontal treatment in the last 6 months, use of medications affecting periodontal tissues within the last 3 months (e.g., antibiotics, anti-inflammatory drugs, anticonvulsants, immunosuppressants, or calcium channel blockers), smoking or recent smoking (within 12 months), pregnancy, extensive prosthetic rehabilitation, orthodontic treatment, blood dyscrasias, alcoholism, and illicit drug use.

### Interventions

After the inclusion in the study, all individuals received detailed information about the etiology of periodontal disease and instructions on oral hygiene, including the use of toothbrushes, dental floss, and interdental brushes, as needed. Baseline standard scaling and root planing (SRP) was performed following the one-stage full-mouth disinfection protocol, without the use of CLX, as reported by (Quirynen et al. 2000). After administering anesthesia using the regional block technique [2% lidocaine solution with epinephrine 1:100,000 (Nova DFL)], the SRP was carried out by an experienced professional (different from the examiner conducting the clinical assessments) using an ulttrasound (Dabi Atlante, Ribeirão Preto, SP, Brazil) and periodontal curettes (Millenium™ Curettes, Golgran, São Paulo, Brazil). The time spent per quadrant was approximately 1 h. Polishing was then performed using a rubber cup and prophylactic paste (Herjos prophylactic paste, Vigodent, Rio de Janeiro, Brazil). No subgingival instrumentation was conducted for the following 45 days.

**Fig. 1:**
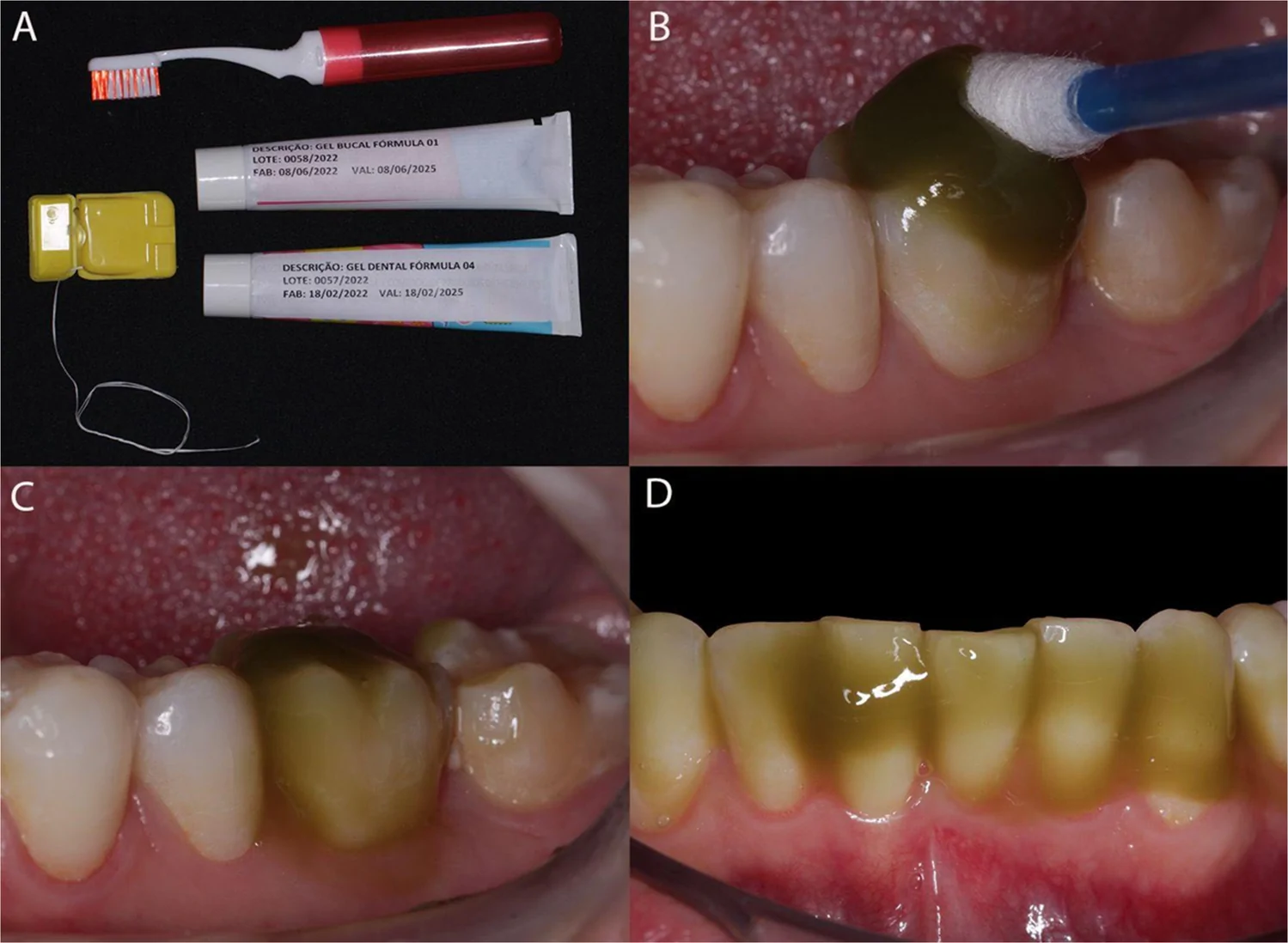
Mouth gel application protocol. (**A**) Oral hygiene kit including toothpaste, dental gel, dental floss, and a toothbrush. (**B**) Supragingival application of the mouth gel to the tooth surface using a cotton swab. (**C**) Final aspect of the tooth after gel application. (**D**) Mouth gel applied by the patient over all tooth surfaces

After the SRP, each individual received a leaflet summarizing the relevant instructions for gel application. Participants were instructed to brush their teeth three times a day using the toothpaste provided by the researchers (Fig. 1A). Following the final brushing of the day, participants were instructed to apply the designated mouth gel supragingivally, over all dental surfaces, using a cotton swab once daily (Fig. 1B-D). The gel was left in contact with the teeth for five minutes, after which the subjects were instructed to expel the excess gel without rinsing their mouths. The composition of the products used is detailed in Table 1. Furthermore, they were instructed not to use any other toothpaste, mouth rinses, or gels during their participation in the study. Participants were also asked to report any signs of irritation, pain, or other adverse effects to the research team for evaluation. To ensure compliance with the use of the formulations, patients were instructed to complete a calendar marking the days and times they used the gels and dental floss. Additionally, daily reminders were sent through a digital communication platform to reinforce adherence.

**Table 1:** Description of the product composition, product manufacturers, and application protocols

Product	Group	Composition	Manufacturer	Application protocol
**Toothpaste**	Vehicle, CLX, and FeTcPc	1450 ppm Fluoride in the form of Sodium Fluoride, Glycerol, Silicon Dioxide, Sorbitol, Water, Sodium Lauryl Sulfate, Flavoring, Macrogol, Sodium Carboxymethylcellulose, Tetrasodium Pyrophosphate, Xylitol, Menthol, Sodium Saccharin Dihydrate, Mica, and Sodium Benzoate	Rabbit, Brazil	Brush your teeth and tongue at least three times a day for approximately 3 min each time
**Mouth gel**	Vehicle	Water, glycerin, sorbitol, hydrated silica, PVP, cellulose gum, flavoring, sodium benzoate, sucralose, phosphoric acid, and sodium hydroxide	Rabbit, Brazil	Apply the gel supragingivally once daily, after the final brushing of the day, to all dental surfaces, and leave it in place for 5 min
**Mouth gel**	CLX	Water, glycerin, sorbitol, hydrated silica, PVP, cellulose gum, flavoring, sodium benzoate, sucralose, phosphoric acid, sodium hydroxide, and **0.12% chlorhexidine digluconate**	Rabbit, Brazil	Apply the gel supragingivally once daily, after the final brushing of the day, to all dental surfaces, and leave it in place for 5 min
**Mouth gel**	FeTcPc	Water, glycerin, sorbitol, hydrated silica, PVP, cellulose gum, flavoring, sodium benzoate, sucralose, phosphoric acid, sodium hydroxide, and **1% iron tetracarboxyphthalocyanine**	Rabbit, Brazil	Apply the gel supragingivally once daily, after the final brushing of the day, to all dental surfaces, and leave it in place for 5 min

### Outcomes

Periodontal clinical parameters were assessed using a Williams millimeter-marked periodontal probe (Hu-Friedy, Chicago, IL, USA) at baseline and at 45 days post-treatment. The parameters evaluated included: probing depth (PD), defined as the distance from the gingival margin to the bottom of the sulcus at six sites per tooth (mesio-buccal, buccal, disto-buccal, mesio-lingual, lingual, disto-lingual); gingival margin level (GML), defined as the distance from the gingival margin to the cementoenamel junction (CEJ) at six sites per tooth, similar to PD; clinical attachment level (CAL), defined as the distance from the CEJ to the bottom of the pocket at six sites per tooth, similar to PD; bleeding on probing (BOP), assessed 30 s after probing and recorded as present or absent at six sites per tooth; and plaque index (PI), defined as the presence or absence of biofilm on the tooth surface, evaluated at six sites per tooth, similar to PD; tooth mobility, assessed using Miller's index (Miller 1950); and furcation involvement, evaluated using Hamp’s classification (Hamp et al. 1975). The percentages of sites with PD and CAL were categorized as follows: PD ≤ 3 mm, PD = 4–5 mm, and PD ≥ 6 mm; and CAL ≤ 2 mm, CAL = 3–4 mm, and CAL ≥ 5 mm.

### Sample size

The sample size calculation was based on the study by Putt et al. (2014), which evaluated the effect of a doxycycline gel on periodontal disease treatment in healthy patients. Considering a clinically relevant difference of a 1 mm reduction in probing depth between groups after treatment and assuming a mean standard deviation of 0.45 mm for this outcome, with a type I error fixed at 0.05 and a beta power of 80%, the minimum sample size required to detect similar outcomes to the aforementioned study was determined to be 15 individuals per group.

### Randomization and allocation concealment mechanism

For randomization, the 47 participants were allocated into the three groups using a computer-generated randomization table with a 1:1:1 allocation ratio. Allocation concealment was ensured by using sealed opaque envelopes containing the treatment assignment from the randomization table for each participant. The envelopes were opened by the operator immediately after SRP completion, and the designated gels were provided to the participants.

### Blinding and examiner calibration

All SRP procedures were executed by the same experienced professional (A. L. M. R.). An investigator masked to the treatment (S.O.P.N.) made the clinical measurements. The examiner did not perform the assigned treatment procedures and was unaware of it. Additionally, patients were blinded to the treatment, as the gels were placed in packets that prevented patients from identifying their contents.

### Statistical methods

The Shapiro–Wilk test demonstrated that the numerical clinical data in this study (number of teeth, PD, GML, CAL, BOP, and PI) followed a normal distribution. Data on mobility and furcation involvement were expressed as event frequencies. A two-way ANOVA, complemented by Tukey’s post hoc test, was used to evaluate the effects of treatment and evaluation period on clinical parameters (number of teeth, PD, GML, CAL, BOP, and PI). The chi-square test was applied to assess the effects of time and treatment on mobility and furcation involvement. Statistical analyses were performed using SPSS version 27. All statistical tests were conducted at a 95% confidence level.

## Results

### Characteristics of the population

**Fig. 2:**
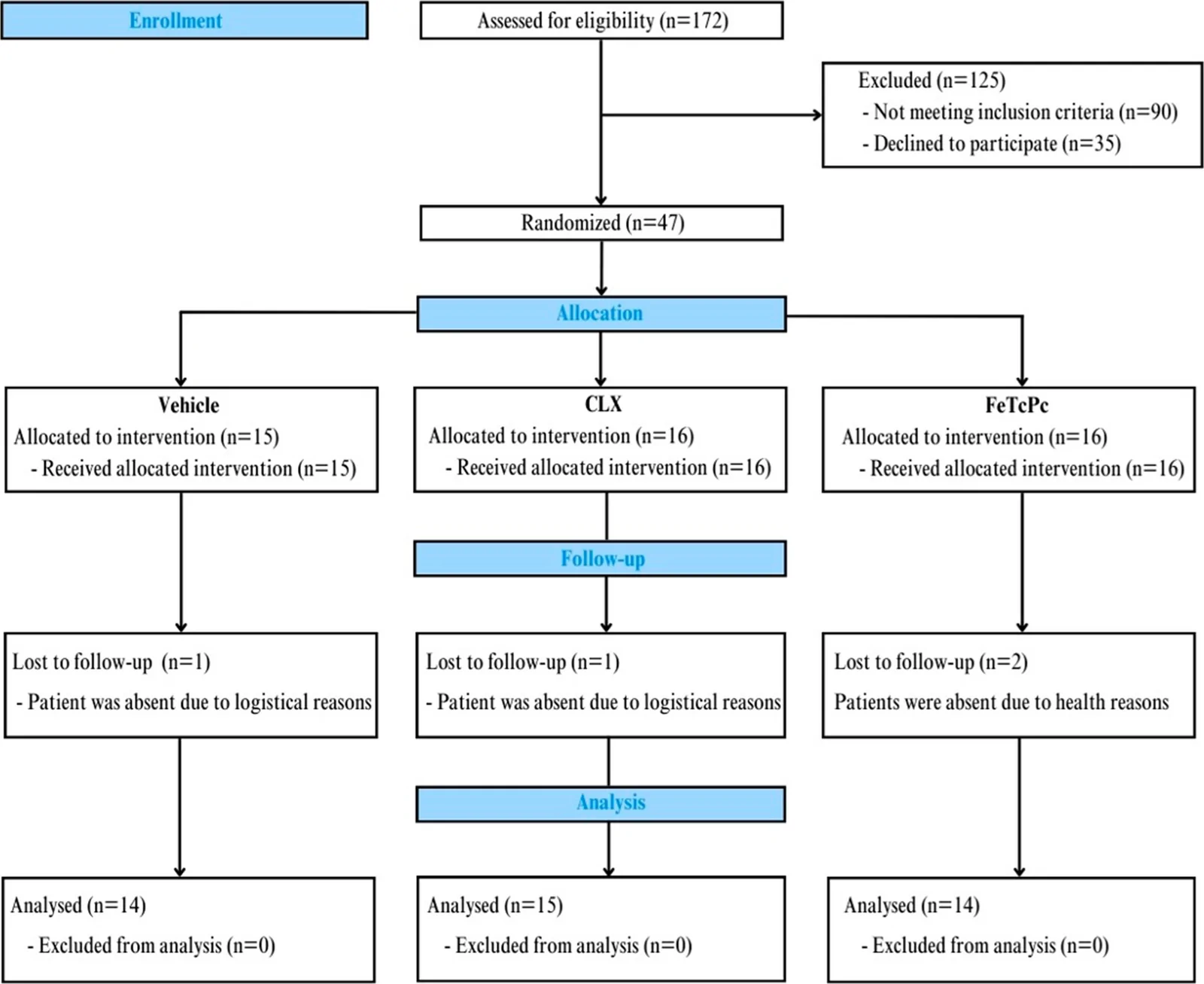
Flowchart of the study

Forty-seven individuals were initially enrolled in the study. At the 45-day follow-up, four participants were lost: one from the Vehicle and CLX group and two from the FeTcPc group (Fig. 2). A total of 43 individuals (30 females and 13 males), with a mean age of 51 years (± 11.4), completed the follow-up. No statistically significant differences were observed between the groups regarding demographic data (age and gender) (Table 2), and no adverse effects were reported by patients following the treatment.

**Table 2:** Demographic characteristics of the population included at baseline

Variables	Vehicle (*n* = 14)	CLX (*n* = 15)	FeTcPc (*n* = 14)
Demographic characteristics			
Age, mean (± SD)	53.6 ± 7.91	50.0 ± 15.4	45.9 ± 8.50
Gender, *n* (%)			
Men	4 (28,5)	7 (46,7)	2 (14,3)
Women	10 (71,5)	8 (53,3)	12 (85,7)

### Clinical outcomes

No statistically significant differences were observed between the study groups in any periodontal clinical parameters at baseline or 45 days after treatment (Table 3). In the intragroup analysis, the mean PD and the percentage of sites with PD ≤ 3 mm and PD = 4–5 mm showed a statistically significant reduction at 45 days in both the vehicle and FeTcPc groups (*p* < 0.05) (Table 3). Additionally, the percentage of sites with BOP and the PI demonstrated a statistically significant reduction after 45 days of treatment in all analyzed groups (*p* < 0.05) (Table 3).

**Table 3:** Means ± SD of periodontal clinical parameters in each evaluation period and treatment group

Groups		Vehicle (*n* = 14)		CLX (*n* = 15)		FeTcPc (*n* = 14)	
Periods		Baseline	45 d	Baseline	45 d	Baseline	45 d
**Number of teeth**		24.0 ± 3.3	24.0 ± 3.3	25.3 ± 4.4	25.3 ± 4.4	24.0 ± 4.3	24.0 ± 4.3
**PD**	**Total (mm)**	**3.14 ± 0.53**	2.84 ± 0.36*	3.24 ± 0.80	3.12 ± 0.51	**3.19 ± 0.37**	2.88 ± 0.38*
	≤ 3 mm (% of sites)	**68.8 ± 15.8**	83.2 ± 10.3*	66.4 ± 20.3	73.9 ± 16.2	**67.6 ± 8.0**	80.9 ± 7.8*
	**entre 4–5 mm (% of sites)**	**23.4 ± 11.0**	13.2 ± 8.8*	20.5 ± 8.7	15.9 ± 8.5	**24.4 ± 7.2**	12.7 ± 6.2*
	≥ 6 mm (% of sites)	5.9 ± 6.5	2.5 ± 4.0	8.4 ± 8.5	6.6 ± 5.7	5.9 ± 6.8	4.0 ± 5.2
**CAL**	**Total (mm)**	3.93 ± 0.86	3.59 ± 0.71	3.91 ± 1.50	3.85 ± 1.32	3.81 ± 0.98	3.57 ± 1.18
	≤ 2 mm (% of sites)	25.7 ± 17.3	27.9 ± 11.7	31.5 ± 19.6	26.7 ± 15.2	27.9 ± 16.0	33.9 ± 20.0
	**entre 3–4 mm (% of sites)**	46.1 ± 13.5	49.1 ± 13.6	42.6 ± 13.0	44.6 ± 18.4	46.1 ± 12.5	43.4 ± 13.6
	≥ 5 mm (% of sites)	28.1 ± 15.3	23.6 ± 15.3	29.3 ± 22.8	27.4 ± 22.8	28.2 ± 21.2	22.6 ± 23.6
**GML (mm)**		0.79 ± 0.45	0.73 ± 0.51	0.69 ± 0.84	0.72 ± 0.92	0.64 ± 0.70	0.70 ± 0.88
**BOP** **(% of sites)**		**82.9 ± 14.7**	33.6 ± 21.1*	**82.1 ± 11.5**	34.8 ± 22.6*	**71.6 ± 19.0**	29.6 ± 17.6*
**PI** **(% of sites)**		**82.4 ± 9.6**	58.8 ± 24.5*	**82.3 ± 11.1**	58.4 ± 22.6*	**78.1 ± 13.2**	59.9 ± 15.8*
**Tooth mobility (number of teeth)**	**I**	8.36 ± 4.63	5.43 ± 2.71	6.40 ± 4.42	3.93 ± 4.08	6.36 ± 3.46	4.29 ± 2.79
	**II**	0.78 ± 1.05	0.71 ± 1.20	0.80 ± 1.32	0.73 ± 1.16	0.35 ± 0.63	0.42 ± 0.64
	**III**	0.00 ± 0.00	0.00 ± 0.00	0.13 ± 0.51	0.20 ± 0.56	0.00 ± 0.00	0.00 ± 0.00
**Furcation involvement (number of sites)**	**I**	0.71 ± 0.72	0.71 ± 0.99	1.00 ± 1.13	0.60 ± 0.98	1.07 ± 1.21	0.71 ± 0.99
	**II**	0.14 ± 0.53	0.21 ± 0.57	0.40 ± 0.63	0.33 ± 0.61	0.35 ± 0.74	0.28 ± 0.61
	**III**	0.00 ± 0.00	0.00 ± 0.00	0.13 ± 0.35	0.13 ± 0.35	0.07 ± 0.26	0.07 ± 0.26

*PD* probing depth, *CAL* clinical attachment level, *GML* gingival marginal level, *BOP* bleeding on probing, *PI* plaque index, *p < 0.05: differences between each follow-up period

## Discussion

In this study, we propose a new mouth gel formulation containing FeTcPc, a phthalocyanine derivative with anti-inflammatory activity, as an adjuvant treatment for patients with periodontal disease. This formulation was previously evaluated in an experimental periodontitis model, where the topical application of a mouth gel containing 1% FeTcPc was shown to block alveolar bone loss (Breseghello et al. 2025). Additionally, 1% FeTcPc reduced the expression of Tnfα, p65 (NF-κB), and Rankl in the gingival tissue (Breseghello et al. 2025). However, the results of this clinical trial demonstrated that the daily supragingival, patient-administered application of the FeTcPc-based mouth gel, as an adjunct to non-surgical periodontal therapy, did not result in statistically significant differences in clinical periodontal parameters compared to the vehicle group after 45 days of treatment, similar to previous clinical trials, which reported limited or no additional clinical benefits from phthalocyanine-based adjunctive therapies despite some improvements in microbiological and inflammatory parameters (Sena et al. 2019; de Araujo Silva et al. 2020; Al-Kheraif et al. 2022a).

One of the distinguishing features of FeTcPc is its reported biological activity even in the absence of external photoactivation (Silva Santos et al. 2021; Bruzadelli et al. 2025), as explored in the present study, suggesting that its effects are not strictly dependent on light-induced excitation and may involve alternative mechanisms within the cellular microenvironment. In contrast, preclinical studies have demonstrated the potential of phthalocyanines combined with photoinduction as an innovative strategy for medical applications, owing to their ability to modulate the inflammatory response (Li et al. 2023). In line with this, Ayaz et al. (2019) observed, in an in vitro macrophage culture, that photoinduced subphthalocyanines with chloro substitution were able to modulate the production of inflammatory cytokines, reducing TNF-α and Interleukin-6 (IL-6) levels. In another study, zinc phthalocyanines subjected to photoinduction (10 min) demonstrated a high capacity to reduce cytokine production, an activity associated with modulation of the p38 pathway (Yuzer et al. 2019). Finally, encapsulated chloroaluminum phthalocyanine combined with photodynamic therapy reduced the inflammatory response (TNF-α levels) and tissue destruction in rats with ligature-induced periodontitis (de Moraes et al. 2017).

Similarly, Seguier et al. (2010) demonstrated that the combination of photodynamic therapy and phthalocyanine had a positive impact on gingival biopsies from patients with chronic periodontitis, leading to a reduction in antigen-presenting cell populations. Likewise, de Araujo Silva et al. (2020) reported that although photodynamic therapy combined with chloro-aluminum phthalocyanine did not yield statistically significant improvements in clinical periodontal parameters when used as an adjunct to SRP, it enhanced the balance between oxidative and non-oxidative agents involved in oxidative stress, a crucial factor in tissue damage and the exacerbation of periodontal inflammation. In addition, Al-Kheraif et al. (2022a) also found that the adjunctive use of photodynamic therapy with chloro-aluminum phthalocyanine and SRP did not significantly improve clinical periodontal parameters compared to SRP alone. However, microbiological analysis revealed a significant reduction in *Porphyromonas gingivalis* and *Tannerella forsythia* levels at 3- and 6-month follow-ups, particularly in smokers. These findings suggest that while chloro-aluminum phthalocyanine-mediated antimicrobial photodynamic therapy may not enhance clinical outcomes, it can modulate the subgingival microbiota, with long-term implications for periodontal health. Sena et al. (2019) also reported that photodynamic therapy with chloro-aluminum phthalocyanine as an adjunct to SRP did not provide additional benefits in reducing probing depth or improving clinical attachment gain.

All these studies share the common approach of using a single application of phthalocyanine directly into the periodontal pocket. However, an increased number of photodynamic therapy applications with other photosensitizers has been associated with greater periodontal benefits, including reductions in probing depth and bleeding on probing (Petelin et al. 2015; Theodoro et al. 2017, 2018), clinical attachment gain (Petelin et al. 2015; Theodoro et al. 2018), decreased residual pockets (Theodoro et al. 2017), and lower levels of periodontal pathogens (Theodoro et al. 2012, 2018; Petelin et al. 2015). In our study, the FeTcPc-containing mouth gel was designed to provide a simple, single daily, non-invasive application that would not cause discomfort, allowing patients to perform the treatment at home, thereby reducing the need for clinical visits and improving adherence through a more practical and cost-effective approach. Considering the formulation properties, the gel presents sufficient fluidity to allow its dispersion toward the gingival margin and potential penetration into the gingival sulcus. However, the single daily application and the limited exposure time may have reduced its effectiveness in fully modulating the inflammatory process.

In addition, no in-office applications were performed directly within periodontal pockets, which may have further limited the delivery of the active compound to deeper sites. It is also important to note that the gel is not a controlled-release system, and therefore, even when applied subgingivally, prolonged retention of the active agent would not be guaranteed. On the other hand, repeated daily applications performed by the patient may increase the cumulative bioavailability of the agent over time compared with single or limited in-office applications (Tan et al. 2021), which could increase treatment cost and reduce patient adherence. Furthermore, the 1% concentration of FeTcPc may not have been sufficient to produce clinical benefits, suggesting that higher concentrations of the bioactive compound should be investigated in future studies. Preclinical toxicity assessments in an alternative model showed no signs of systemic toxicity within 72 h of FeTcPc administration (Breseghello et al. 2025).

In this study, the efficacy of the FeTcPc-based mouth gel was evaluated in patients with mild to moderate periodontal disease, which may also have limited the product's pharmacological effects on the inflammatory process. Patients with less severe disease generally respond well to basic non-surgical periodontal treatment (Cobb and Sottosanti 2021), potentially masking any additional benefits provided by the FeTcPc-containing adjunctive gel. Supporting this, a previous study combining scaling and root planing (SRP) with photoinduced chloro-aluminum phthalocyanine demonstrated significant improvements in clinical parameters, cytokine levels, and oral health-related quality of life in individuals with stage III periodontitis associated with Parkinson’s disease (Al-Kheraif et al. 2023). This could also explain why the 0.12% CLX mouth gel did not exhibit significant pharmacological benefits compared to the vehicle and FeTcPc groups in the present study.

CLX is a bisbiguanide with antiplaque activity used in dentistry, both therapeutically and prophylactically, and remains active for several hours due to its high affinity for oral surfaces (Poppolo Deus and Ouanounou 2022). Studies have shown that using a mouthwash containing 0.12% CLX as an adjunct to scaling and root planing therapy can provide additional reductions in periodontal disease compared with conventional treatment alone (Faveri et al. 2006; Feres et al. 2009, 2012; Fonseca et al. 2015). However, in all these studies, patients used CLX as a mouth rinse twice daily, whereas in our study, it was applied once daily directly onto the tooth surface. The use of CLX as a mouth rinse disrupts periodontal pathogen reservoirs that are not reached by scaling and root planing, such as the tonsils, tongue, saliva, and oral mucous membranes, which may positively influence the recolonization of recently scaled pockets (Faveri et al. 2006). This broader antimicrobial effect was not achievable with the single daily application restricted to the tooth surface in our study, which may have contributed to the lack of significant clinical benefits observed.

It is important to highlight that patients with systemic conditions or smokers could benefit more from the anti-inflammatory effects of the FeTcPc-based mouth gel, as their immune response is often more compromised compared to generally healthy patients included in this study (Kinane et al. 2017; Leite et al. 2018; Sanz et al. 2020). These populations typically exhibit higher levels of inflammation and a reduced capacity for periodontal disease recovery (Kinane et al. 2017), making the gel’s adjunctive anti-inflammatory properties particularly valuable. Supporting this, a study by Al-Kheraif et al. (2022b) investigating photodynamic therapy with chloro-aluminum phthalocyanine in combination with SRP in patients with stage II chronic periodontitis found that smokers in the combined therapy group experienced a significant reduction in bleeding on probing at 6 months. While probing depth and clinical attachment levels improved across all groups, only smokers in the combined therapy group exhibited a significant decrease in *Porphyromonas gingivalis* and *Tannerella forsythia* levels at both 3- and 6-month follow-ups. Additionally, TNF-α levels decreased significantly in smokers in both treatment groups, whereas IL-1β reduction was observed only in non-smokers at 6 months in the combined therapy group. These findings highlight the need for further research focusing on these specific patient groups to determine whether the FeTcPc-based mouth gel could provide therapeutic benefits in more challenging clinical conditions.

Several limitations should be considered when interpreting the results of this study. The inclusion of patients with mild to moderate periodontal disease, who typically respond well to basic periodontal therapy, may have further masked any adjunctive benefits of the FeTcPc gel. Another limitation was the lack of a longer follow-up period, such as 3 months, which prevented the assessment of the long-term maintenance of clinical improvements. However, the 45-day evaluation was intentionally selected to capture the early healing phase following SRP and to assess the immediate efficacy of the adjunctive treatment under controlled conditions, minimising the influence of long-term behavioral and maintenance-related variables. Although the components of the vehicle are not known to exert significant anti-inflammatory effects on periodontal tissues, the absence of a control group receiving SRP alone may be considered a limitation, as it does not allow complete exclusion of any potential residual influence of the vehicle on the observed outcomes.

Additionally, the absence of photoactivation, which may influence the biological activity of FeTcPc, should also be considered when interpreting the findings. Moreover, no microbiological or immunological analyses were performed, which limits the understanding of the intervention potential effects on subgingival microbiota and inflammatory mediators. In addition, the supragingival, patient-administered application protocol adopted in this study may have limited the direct delivery of the active compound into periodontal pockets, potentially reducing its effectiveness in deeper sites. Future studies should investigate alternative delivery methods, including subgingival or combined application protocols, as well as formulations with sustained-release properties, in order to optimize therapeutic outcomes while maintaining clinical feasibility.

## Conclusion

The findings of this study indicate that the FeTcPc-based mouth gel, when used as an adjunct to non-surgical periodontal therapy, did not significantly improve clinical periodontal parameters compared to CLX or the vehicle group. However, these investigations provide valuable insights into the potential role of FeTcPc in periodontal disease management, highlighting the need for further research to optimize its formulation and therapeutic application.

## Data Availability

The data supporting this study's findings are available from the corresponding author upon reasonable request, with certain restrictions due to privacy or ethical considerations.
